# The spatiotemporal variations of microbial community in relation to water quality in a tropical drinking water reservoir, Southmost China

**DOI:** 10.3389/fmicb.2024.1354784

**Published:** 2024-05-06

**Authors:** Di Wu, Ying Zou, Juan Xiao, Ling Mo, Sovan Lek, Bo Chen, Qiongyao Fu, Zhiqiang Guo

**Affiliations:** ^1^School of Life and Health Sciences, Key Laboratory of Tropical Biological Resources of Ministry of Education, State Key Laboratory of Marine Resource Utilization in South China Sea, Hainan University, Haikou, China; ^2^Institute of Environmental Sciences, Haikou, Hainan Province, China; ^3^Laboratoire Evolution & Diversité Biologique, Université Paul Sabatier, Toulouse, France; ^4^National Health Commission of the People's Republic of China Key Laboratory of Control of Tropical Diseases Control, School of Tropical Medicine, Hainan Medical University, Haikou, Hainan, China

**Keywords:** Songtao Reservoir, microbial composition, spatiotemporal distribution characteristics, environmental factors, 16S rRNA

## Abstract

It is well-known that water quality has great significance on microbial community composition in aquatic environments. In this study, we detected water column indicates the microbial community composition of nine sampling sites over two seasons using Illumina TruSeq sequencing in Songtao Reservoir, Hainan Province, Southmost China. The study indicated that the dominant phylum was Actinobacteria, Proteobacteria, Bacteroidetes, and Cyanobacteria. The diversity parameters showed that the microbial community composition had significant spatiotemporal variations, including the significantly higher Shannon index and Simpson index upstream than those midstream and downstream. Besides, there were significantly higher Chao1 index, Shannon index, and Simpson index in winter than in summer. Principal coordinates analysis (PCoA) showed the microbial structural composition had significant seasonal differences. The results of microbial community composition further revealed that the eutrophication level upstream was higher than that of midstream and downstream. The redundancy analysis (RDA) diagram indicated that the abundance of microbiology species significantly correlated with temperature, total phosphorus, Se, and Ni. Furthermore, the mantel's test showed that the temperature and total phosphorus significantly affected the community composition of archaea and bacteria. Overall, our finding here partially validated our hypothesis that the spatiotemporal variations of microbial community composition are significantly related to nutrients, physicochemical factors and metals, which has been unknown previously in tropical drinking waterbodies. This study substantially contributed to understanding of the composition of microbial community in tropical drinking water reservoirs and the main environmental driving factors in tropical zones. It also provided a reference for the management of reservoir operation to ensure drinking water safe.

## 1 Introduction

Approximately 3% of the world's water constitutes freshwater, but only 0.3% of this freshwater, found in lakes and rivers, is directly accessible to humans (Musie and Gonfa, 2023). And there are still problems of uneven distribution and water pollution, which lead to water quality and biosecurity of drinking water have been widely addressed (Gunnarsdottir et al., 2020). The water quality is closely affected by the composition of the microbial community in the water (Wani et al., 2021). The composition of microbial communities will rapidly vary, once their habitats have changed slightly. Some studies have shown that there was a high concentration of chemical oxygen demand when the abundance of Bacillus and Firmicutes was high as well (Niu et al., 2019; Chen et al., 2020). Springe et al. (2021) found that the counts in microbial indicators were affected by physicochemical parameters such as turbidity, temperature, and pH. In addition, microbial community not only reflect the composition and function of aquatic ecosystems (Jiang et al., 2021; Ajaya et al., 2024), but are also an important part of the biological community of the reservoir ecosystem and plays an important role in material cycle, energy flow, and information transfer. The detritus formed by the microbial community and their organic substrates is an important bait resource for fish in the reservoir (Zhao and Wei, 2018). The diversity, quantity, and special species of microbial community are often used as indicators for water quality. For instance, the total coliforms, Thermotolerant coliforms, and *Escherichia coli* were used as key parameters in China Surface Water Environmental Quality Standard GB3838–2002 (Cui et al., 2015). It's widely recognized that certain waterborne microbial communities pose risks to human health, such as pathogenic microbial community and some cyanobacteria which can release neurotoxins, hepatotoxin, cytotoxin, and endotoxin (Buratti et al., 2017). Therefore, it is crucial to investigate the microbial community composition and spatiotemporal distribution characteristics in drinking water reservoirs.

It is well known that environmental variables (including total nitrogen levels, pH values, and hydrodynamic forces, etc.) play a crucial role in shaping the composition of microbial communities (Read et al., 2015). In several polluted waters, the microbial community composition is also affected by contaminants (Song et al., 2021). Niu et al. (2019) found that the compositions of the microbial community varied along the TGR (Three Gorges Reservoir), and the main environmental factors impacting the composition of the microbial community included ${\text{PO}}_{4}^{3-}$, and COD (chemical oxygen demand). Previous work also found that the composition variation in microbial community was greater in summer than in winter (Jiang et al., 2021). In the Yangtze River, for instance, a previous study reported that mid-February to early March was a high risk period for algal blooms and the main contribution factors for algal blooms included water level, TP and water temperature (Cheng et al., 2019). The high salinity and nutrients were also found to induce dramatic declines in microbial species diversity (Tang et al., 2021). When the algae grew excessively, it often led to the production of toxins and decreasing of DO in the water. Thus, the survival of other aquatic organisms and water quality could be affected in most cases (Misra, 2011). Therefore, clarification of microbial community composition characteristics and their relationship with environmental factors has a great significance for the management of water quality, evaluation of water safety, and risks, which is extremely critical for the drinking water source reservoirs.

As the largest reservoir in Hainan Province, the Songtao Reservoir serves as a vital freshwater source and fulfills various functions including irrigation, flood control, aquaculture, and navigation (Mo et al., 2016). Previous studies showed that pollutants are more likely to be deposited in reservoirs than in rivers, lakes, and other water bodies due to their slow water velocity and long retention time (Zamani et al., 2018). Thus, there was great concern over the water environment in Songtao Reservoir due to its ecological sensitivity and environmental significance. However, since the construction of Songtao Reservoir in 1963, there has been no research on the microbial community.

In this research, our hypothesis was that the variations of water quality across time and space resulted in significant differences in microbial community composition in a tropical drinking water reservoir. Specifically, we examined the distribution of microbial community and investigated the factors influencing in Songtao Reservoir. Our approach involved employing high throughput sequencing to analyze the spatial and temporal diversity as well as the abundance of microbial community. We aimed to reveal the spatial and temporal fluctuations of microbial community in a tropical drinking water reservoir. Additionally, we identified the primary environmental factors that impact microbial community and aimed to offer valuable insights for the management of reservoir microbial community and the safety of drinking water.

## 2 Materials and methods

### 2.1 Sample collection

**Figure 1:**
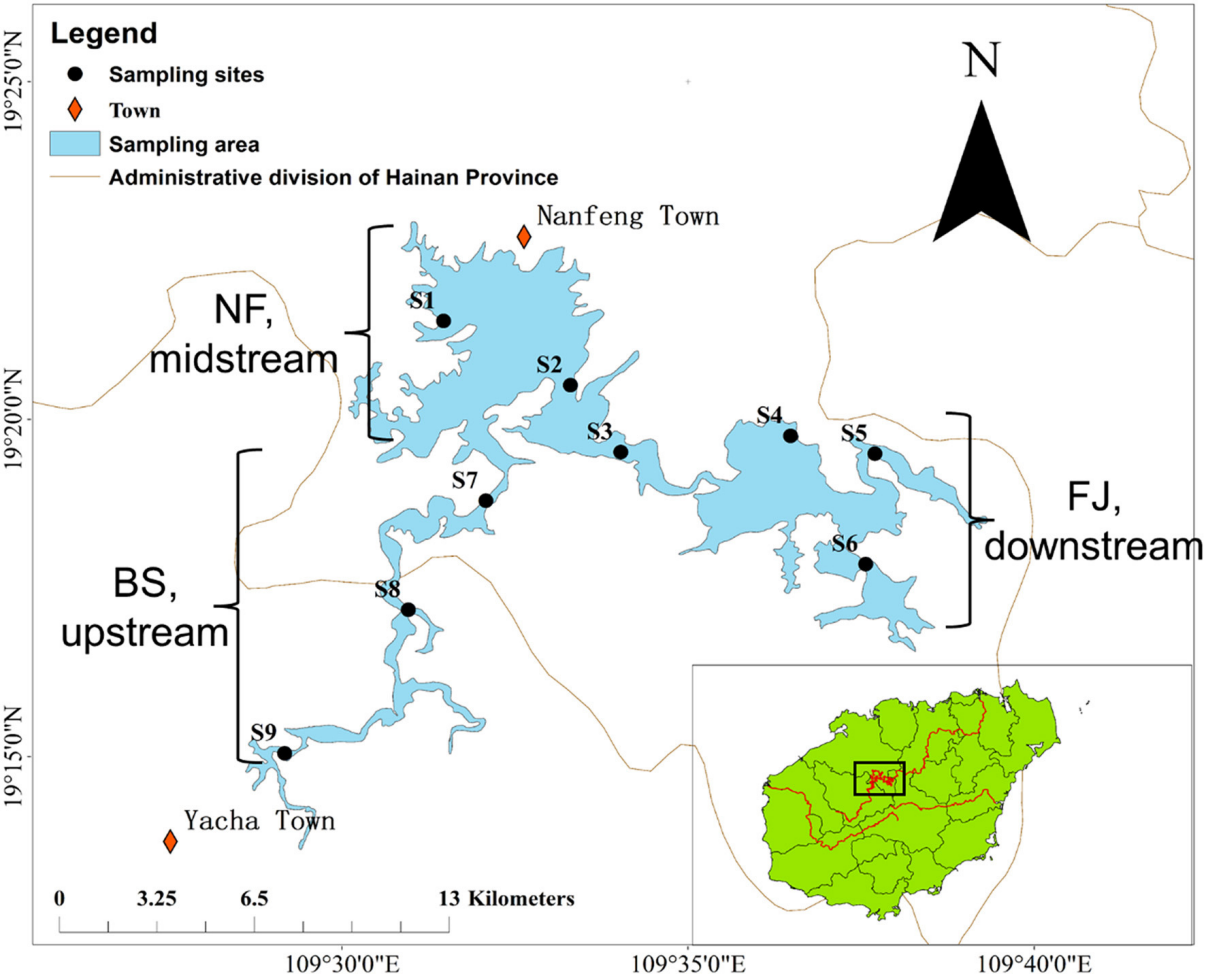
Location of sampling sites in Songtao Reservoir. BS, NF and FJ represents upstream (S7-S9), midstream (S1-S3) and downstream (S4–S6), respectively.

Samples were collected in June (summer) and December (winter) 2020 during the day (10 AM to 17 PM) from the Songtao Reservoir of Hainan province, China (Figure 1). In this study, a total of 9 sampling sites were selected, namely BS (upstream, S7-S9), NF (midstream, S1-S3) and FJ (downstream, S4-S6). We used the sterile sample glass bottles (1 L) to collect water samples at a depth of 10–20 cm below the water surface. Six samples were collected at each sampling point (every samples were obtained at intervals of 3 m) and 1 L of water was filtered per sample. The water samples of microbial were transported to a laboratory at 4°C for filtration.

### 2.2 Environmental factors analysis

pH, EC (electrical conductivity, mS cm^−1^), DO (dissolved oxygen, mg L^−1^), Tem (temperature, °C), TDS (total dissolved solids, g L^−1^), ORP (oxidation-reduction potential, mV), and Tur (turbidity, NTU) were determined by multi-purpose meters (YSI and turbidity meter) in the field. TN (total nitrogen, mg L^−1^), TP (total phosphorus, mg L^−1^), and COD (permanganate index, mg L^−1^) were determined by spectrophotometric methods (The State Bureau of Quality and Technical Supervision, 1989a,b,c). All chemical analyses were conducted in triplicates. The water samples used were transported to the laboratory at 4°C. Prior to analysis, the water samples were filtered through 0.45 μm porous polycarbonate membranes (Collins, Shanghai, China).

The metal concentrations (Cr, Mn, Ni, Cu, Se, Cd, and Ba) in water were determined in this study by ICP-MS (Thermo scientific iCAP RQ). Firstly, the water samples were filtered through a 0.45 μm membrane. And then nitric acid was added until its concentration reached 2%. The internal standard method was used for the calibration method (Sc, Ge, In, Bi as internal standard elements), and the recovery rate was 98% to 109.8%. The concentration of metal elements in water was expressed by μg L^−1^.

### 2.3 Microbial community analysis

The water samples were filtered using a 0.22 μm membrane and stored at −80°C. All microbial community analysis was conducted in sextuplicates. The CTAB method was used to extract the total genome DNA from samples. A total of 1% agarose gels were used to monitor DNA concentration and purity. And the sterile water was used to dilute DNA to 1 ng μL^−1^. We used the specific primer 341F(5′-CCTAYGGGRBGCASCAG-3′) and 806R(5′-GGACTACNNGGGTATCTAAT-3′) to amplified 16S rRNA genes of distinct regions (16S V3-V4). TruSeq^®^ DNA PCR-Free Sample Preparation Kit (Illumina, USA) was used to generate the sequencing libraries and index codes were added. Then, the Qubit@ 2.0 Fluorometer (Thermo Scientific) was used to assess the library quality. Finally, the library was sequenced on an Illumina NovaSeq platform and 250 bp paired-end reads were generated. The 16S rRNA sequence is deposited at NCBI with the BioProject ID PRJNA933804 and SRA ID SRP422163.

### 2.4 Data processing

To study the feature table of the operational taxonomic unit (OTU), the initial step involved converting raw data FASTQ files into a format compatible with the QIIME2 system using the qiime tools import program. Subsequently, sequences from each sample were demultiplexed, underwent quality filtering, trimming, and de-noising. Following this, the sequences were merged, and chimeric sequences were identified and eliminated using the QIIME2 dada2 plugin (Behera et al., 2021). We used the classification criterion of similarity ≥ 99% to divide the OTU of (similarity of species level by default) (Bokulich et al., 2018). We used the OTUs, Chao1 index, Shannon index, and Simpson index to assess the microbial diversity within an individual sample. The principal coordinate analysis (PCoA) and analysis of similarities (ANOSIM) were used to visualize the structural variation of microbial community across samples (beta diversity) (Vázquez-Baeza et al., 2013). The redundancy analysis (RDA) and mantel's test were used to assess environmental factors driving microbial community composition. All data were log (X+1) transformed before being analyzed.

Present partial least squares structural equation modeling (PLS-SEM) was used to evaluate the direct and indirect relationships between microbial OTUs and season (summer and winter), region (upstream, midstream, and downstream), physicochemical factors (Tem, DO, C, TDS, pH, ORP, Tur), nutrients (TN, TP, COD), and metals (Cr, Mn, Ni, Cu, Se, Cd, and Ba). The season and region were given assignments (1: summer, 2: winter, and 1: upstream, 2: midstream, 3: downstream, respectively). SmartPls 4.0 was used to construct the model. Firstly, we constructed a structural model consisting of six main latent variables, namely Season, Region, Physicochemical factors, Nutrients, Metals, and Microbial, and established relationships among the latent variables. Secondly, all observed variables were then imported and the model was fitted. Finally, the model was optimized by eliminating the indicators with low reliability and validity through the fitting results. The results of reliability and validity was shown in Supplementary Table S3. The detailed information on the constructive model referenced by Wen and Li (2019).

One-way ANOVA was used for the inter-group difference with *P* < 0.05 of significant difference. The significant difference in data was detected by IBM SPSS statistics 20. The R language program and GraphPad Prism 8.0 were used to draw heat maps, bar plots, and other figures. The figure of sampling sites was drawn by Arcgis 10.7 and Google Earth.

## 3 Results

### 3.1 Microbial alpha diversity and operational taxonomic units

**Figure 2:**
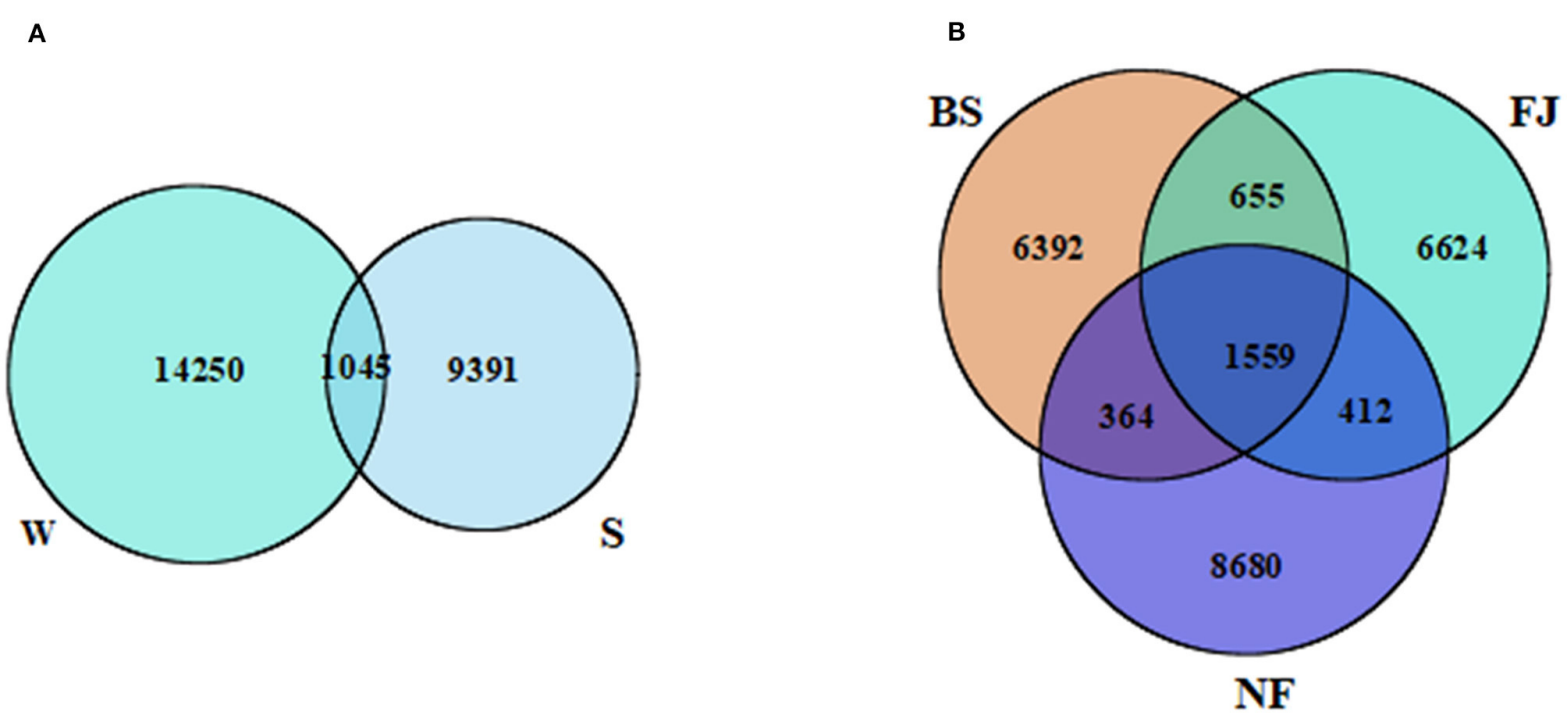
Venn diagram presenting the spatiotemporal distribution characteristics of OTUs. **(A, B)** Represents the temporal and spatial distribution characteristics, respectively. W, winter; S, summer; BS, upstream; NF, midstream; FJ, downstream.

According to the sequencing results, there were significant variation in the microbial community across different periods and regions (Figure 2). A total of 24,686 OTUs were identified in all samples. Notably, distinct OTUs were identified during summer (9,391, 38.04%) and winter (14,250, 57.73%), with only a minimal overlap (1,045, 4.23%) between the two seasons (Figure 2B). Additionally, variations in OTUs number were observed in different regions (Figure 2A). The largest number of OTUs was recorded in NF (11,015, 44.62%), followed by FJ (9,250, 37.47%), and the lowest in BS (8,970, 36.34%). The number of OTUs shared by the three regions was 1,559 (6.32%). The unique OTUs number of NF, FJ, and BS were 8,680 (35.16%), 6,624 (6.83%), and 6,392 (25.89%), respectively.

**Figure 3:**
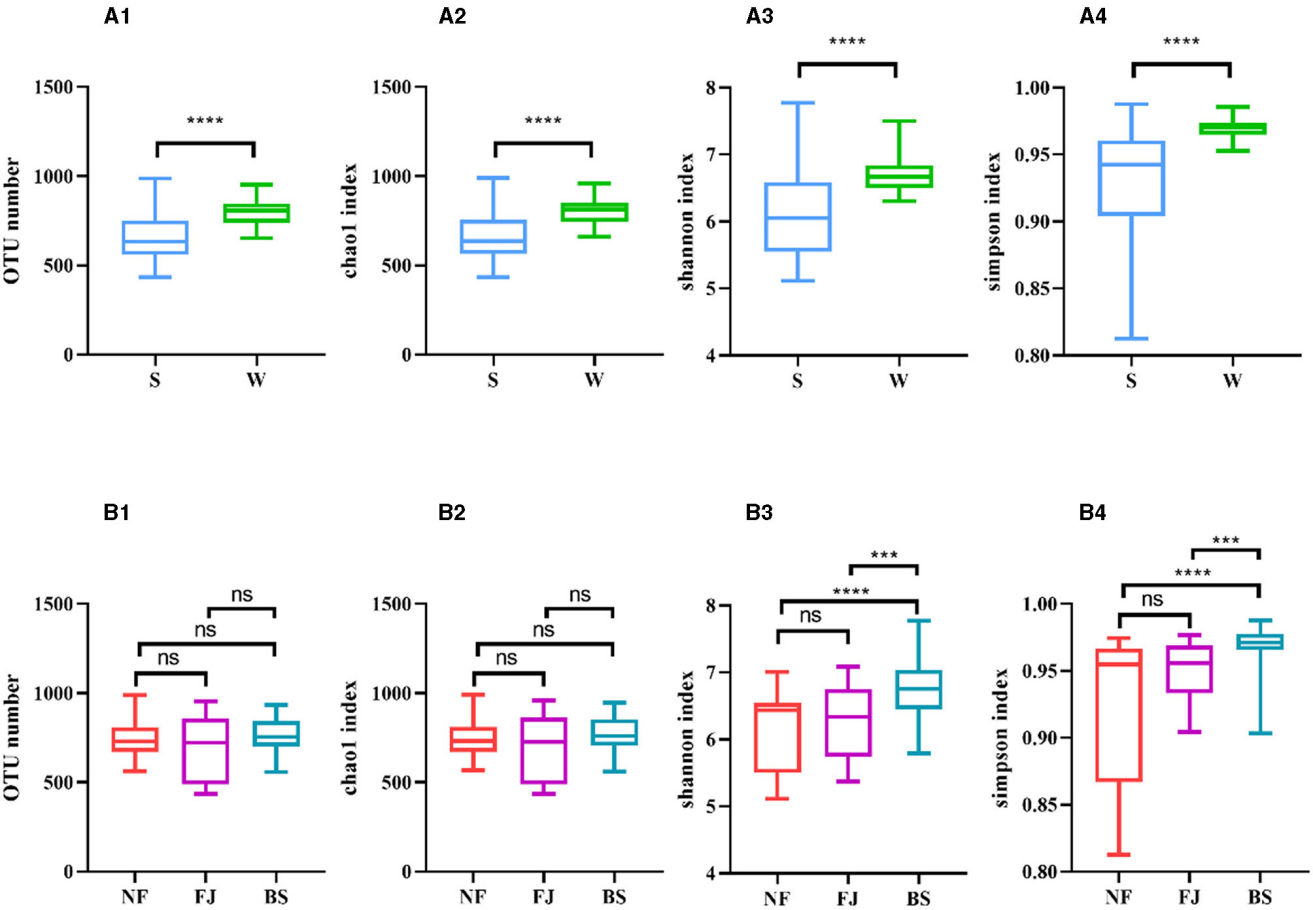
Number of OTUs and alpha diversity indexes in each sample. **(A, B)** Represent classification by time and space, respectively. Number of OTUs (1), Chao1 index (2), Shannon index (3), Simpson index (4). ns, no significant; ***P < 0.001 and ****P < 0.0001.

As shown in Figures 3A2, A3, A4, the Chao1 index, Shannon index, and Simpson index in winter exhibited significantly higher values compared to summer (*P* < 0.01). Regarding spatial distribution characteristics, while the number of OTUs and the Chao1 index remained relatively stable, while the alpha diversity indexes in BS were significantly higher than the other two regions (*P* < 0.001, Figures 3B1, B2, B3, B4).

### 3.2 Compositions of microbial community

**Figure 4:**
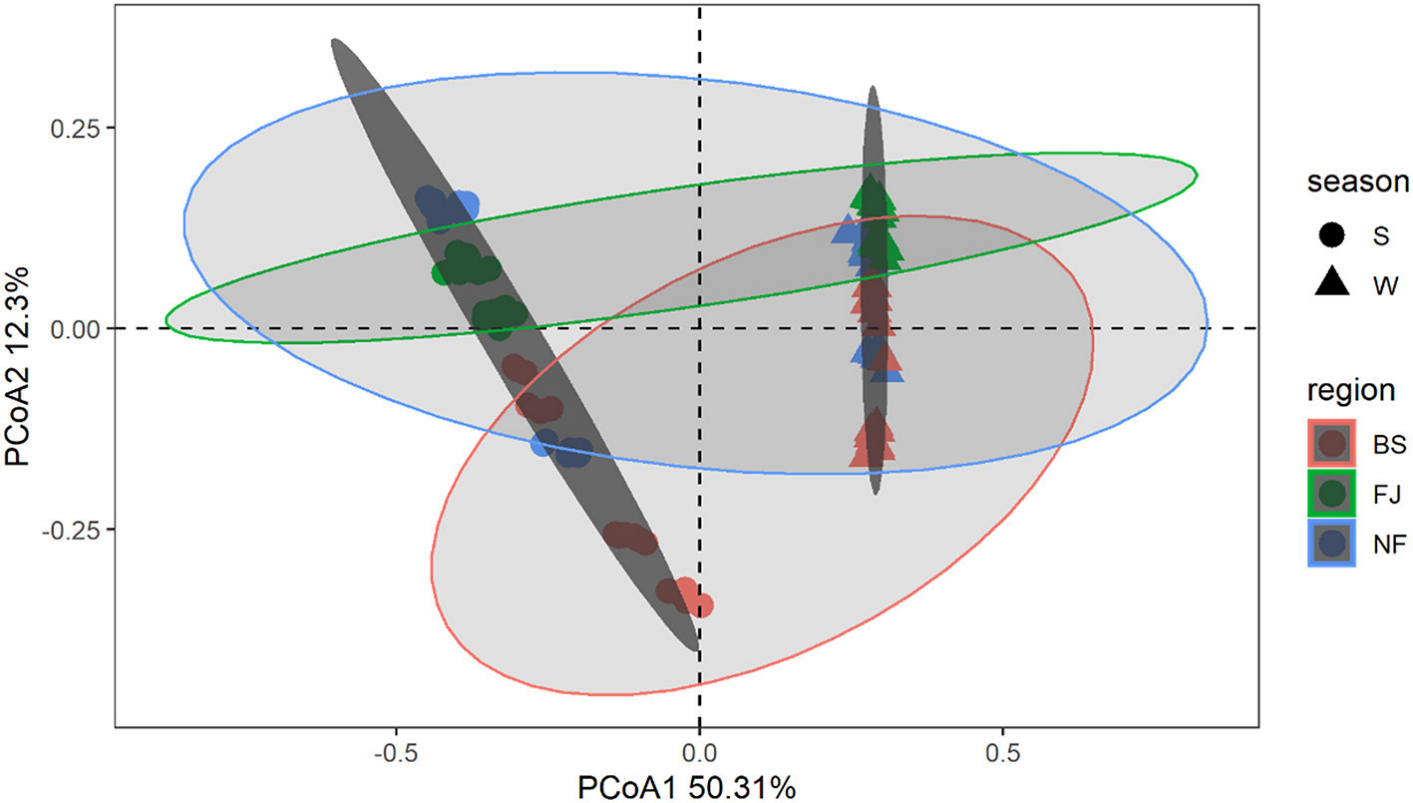
PCoA plot of microorganisms for each sample in Songtao Reservoir. Different color represents different regions (red is BS, blue is NF, green is FJ). Different shape represents different season (circles are summer, triangles are winter). Darker ellipses and lighter ellipses are sorted by time and space, respectively.

In Figure 4, we used the Bray-Curtis distance to study the similarity of all samples in PCoA. It is possible to calculate the quantitative characteristics of the different species composition of a biological sample species by the Bray-Curtis distance. The microbial community composition in the data was explained by PC1 (50.31%) and PC2 (12.30%) (Figure 4). Notably, the distribution of microbial communities at the same location varied significantly across different seasons. Meanwhile, there was a significant spatial difference between BS and FJ in the microbial community composition distribution. Overall, the seasonal variation in microbial community was larger than the spatial variation. ANOSIM results (Supplementary Figure S1) further confirmed that there were significant spatial and temporal differences in the microbial composition of the Songtao Reservoir.

### 3.3 Variations of microbial abundance

#### 3.3.1 Phylum level diversity

**Figure 5:**
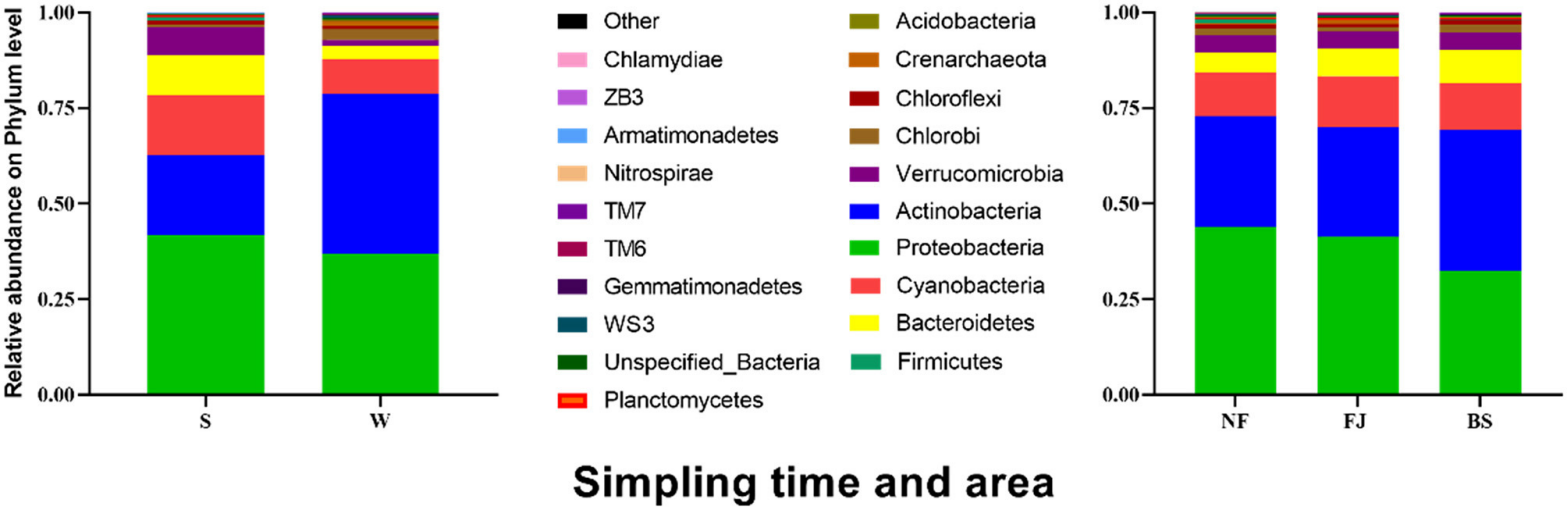
Relative abundance of the main microbial community (the top 20) at phylum level in Songtao Reservoir.

At the phylum level, we observed temporal and spatial variation characteristics and abundance of dominant populations in Songtao Reservoir, as depicted in Figure 5. There were obvious variations in the temporal changes of the dominant microbial populations. The dominant species were Proteobacteria (41.64%), Actinobacteria (21.16%), Cyanobacteria (15.51%), Bacteroidetes (10.55%), Verrucomicrobia (7.35%) in summer, and were Actinobacteria (41.73%), Proteobacteria (36.84%), Cyanobacteria (9.23%), Bacteroidetes (3.43%), Chlorobi (2.74%) in winter. LEfSe was used to analyze the microbial abundance differences at the phylum level between the two seasons (Supplementary Figure S2) (LDA SCORE > 2 as the standard). Actinobacteria in summer was significantly lower than that in winter, while Proteobacteria, Verrucomicrobia, Cyanobacteria, and Bacteroidetes in winter were significantly lower than that in summer (*P* < 0.001). Moreover, we observed similarities in microbial community compositions between NF and FJ, including Proteobacteria (43.95% and 41.33%), Actinobacteria (28.94% and 28.58%), Cyanobacteria (11.42% and 13.42%), while BS exhibited a different composition with Actinobacteria (36.83%), Proteobacteria (32.44%), and Cyanobacteria (12.27%). The results of the comparison in microbial community relative abundance showed that the percentage of Proteobacteria in NF was significantly higher than the other two reservoir regions, and the Actinobacteria in BS was highest among three regions.

**Figure 6:**
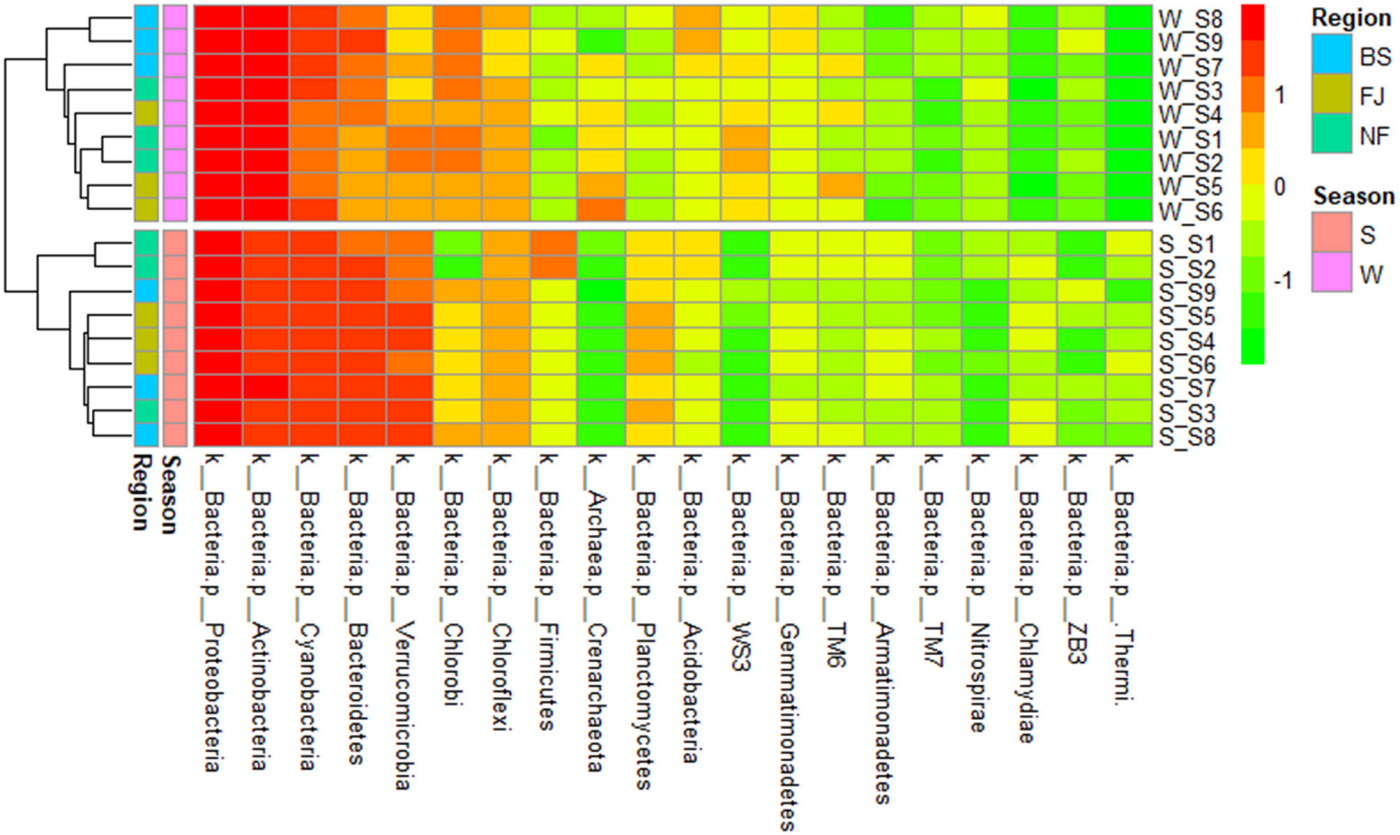
Cluster heatmap of the main abundance of microbial composition (the top 20) at phylum (in the heatmap, moving from red to green represents decreasing relative abundance of microbial composition; for cluster analysis, the same color represents clustered into one group).

We clustered the absolute abundance of all samples and constructed a cluster tree to determine the similarity between different samples (Figure 6). The clustering results showed that the microbial community composition of all sampling sites had significant seasonal differences, with distinct groupings observed for summer and winter. However, within the same season, the variation in microbial community composition across all sampling points was minimal, which indicated that the seasonal variation in microbial communities outweighed spatial variation.

#### 3.3.2 Genus level diversity

**Figure 7:**
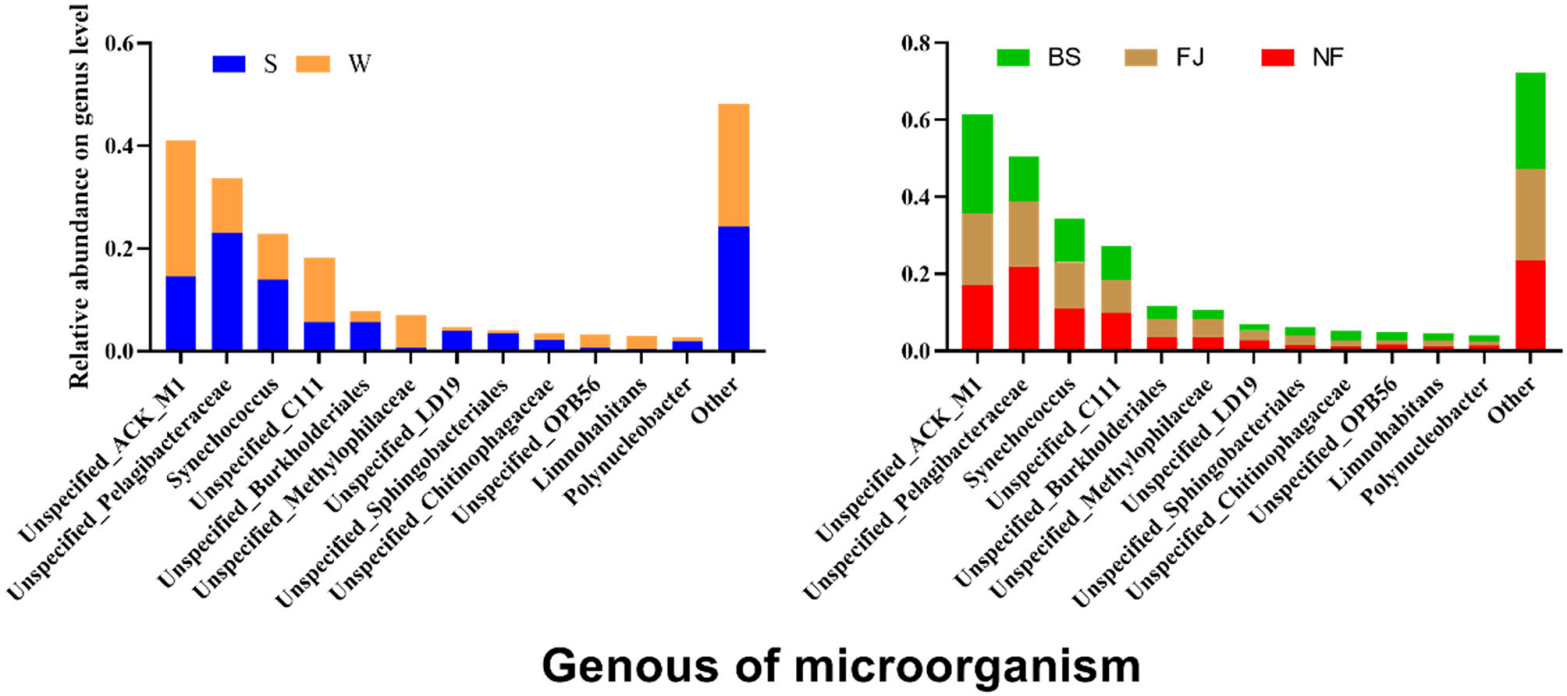
Relative abundance of the main microbial composition (top 12) at genus.

We conducted a comparison of microbial community compositions at the genus level (Figure 7). Our analysis revealed that a total of 12 species microbial community with the highest relative abundance at the genus level accounting for 75% of the total abundance, including *Actinobacteria, Pelagibacteraceae, Synechococcus*, etc. Furthermore, the relative abundance of *Actinobacteria, Acidimicrobiales, Methylophilaceae*, and *Methylophilaceae* exhibited significantly higher relative abundances in winter compared to summer (*P* < 0.01), whereas relative abundances of *Pelagibacteraceae, Synechococcus, Burkholderiales, LD19, Sphingobacteriales*, etc were significantly lower in winter than in summer (*P* < 0.05). Noteworthy spatial differences were also observed, with *Actinomycetales* comprising 25.91% of the microbial community in BS, significantly higher than in NF (17.07%) and FJ (18.48%). Conversely, *Pelagibacteraceae* accounted for 11.45% in BS, significantly lower than in NF (21.93%) and FJ (17.01%).

The LEfSe was also used to analyze the differences of all classified microbial communities at genus (LDA SCORE > 4 as the standard) (Supplementary Figure S3). We identified 19 biomarkers in winter and 13 in summer. Spatial differences revealed 12 biomarker species, with 9 in BS, 2 in FJ, and 1 in NF. Interestingly, all *Cyanobacteria* were significantly lower in winter than in summer, suggesting the cyanobacterial bloom was more likely to outbreak in summer than winter in Songtao Reservoir.

### 3.4 Relationship between environmental factors and microbial community

**Figure 8:**
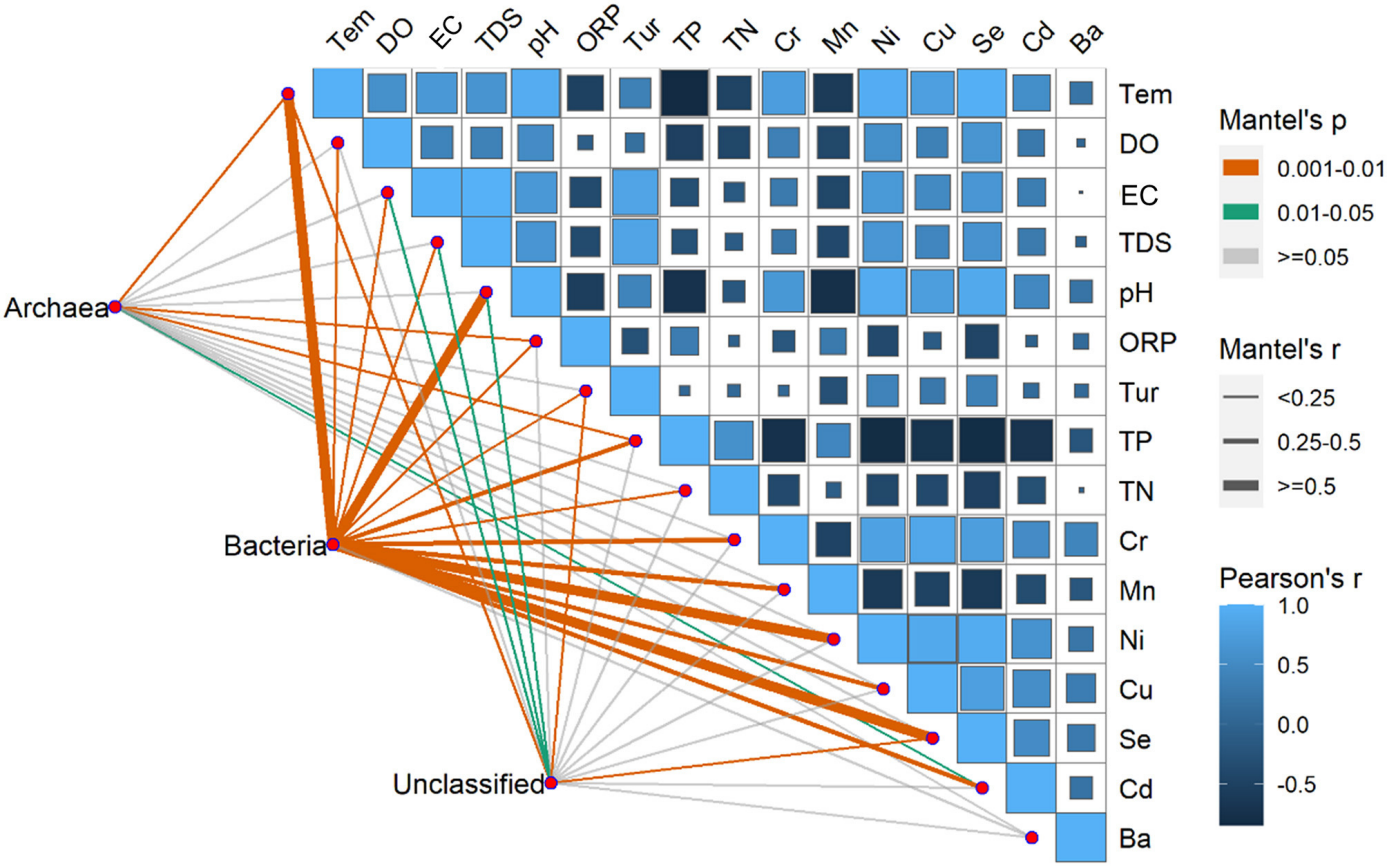
Pearson's correlation coefficients of the main 16 environmental factors, the three microorganism groups (Archaea, Bacteria, unclassified), and microorganism community composition using mantel's tests. Square size represents the significant correlations of *P*. The shade of the color represents the value of the correlation coefficients. Line width corresponds to Mantel's r statistic for the corresponding distance correlations, and line color denotes the statistical significance based on 9999 permutations.

The environmental factors exhibited significant variations over time, with pH, EC, DO, Tem, Ni, Se, and Cd registering significantly higher levels during summer compared to winter. Conversely, ORP, TN, TP, and Mn exhibited significantly higher levels during winter than summer. Furthermore, in terms of spatial distribution, the BS region exhibited peak values solely for TN, TP, COD, and TDS (Supplementary Table S1). Moreover, Pearson's correlation showed that there were correlations between environmental factors except for the Ba, TN, and Tur (Figure 8). Significant correlations between the Tem and the Se and Ni were found, especially. Pearson's correlation coefficients were < 0.90 among Se, Ni, and Tem; among Cu, Se, and Ni and between pH and Se.

**Figure 9:**
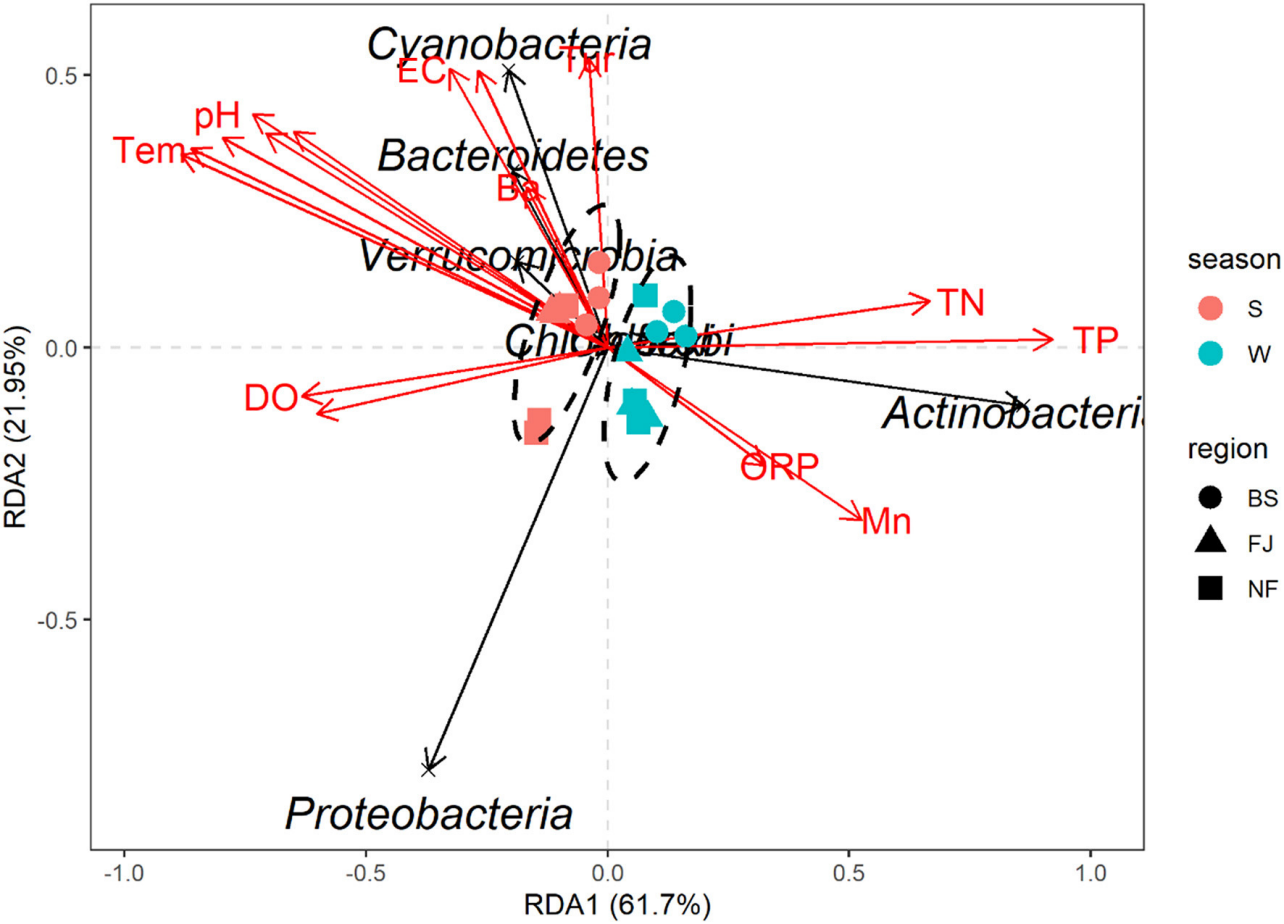
Redundancy analysis diagram at the phylum level. Species in the RDA chart are represented by black arrows. The arrows in red represent quantitative environmental factors. The length of the arrows represents the impact of environmental factors on species. The angles formed by the arrows of environmental factors represent positive or negative correlations. The ellipses are sorted by time.

In this study, RDA was used to study the impact of environmental factors (pH, Tem, DO, TN, TP, Cr, Mn, etc, Supplementary Table S2) on microbial community composition (Figure 9). The explanation amount of RDA1 and RDA2 were 61.70% and 21.95%, respectively. The highest correlation coefficient with RDA1 was TP (*r* = 0.9999), followed by TN (*r* = 0.9949), DO (*r* = −0.9933), Cd (*r* = −0.9845), Se (*r* = −0.9206), Tem (*r* = −0.9135), Ni (*r* = −0.8931), Cr (*r* = –0.8669), pH (*r* = −0.8567), Mn (*r* = 0.8496), Cu (*r* = −0.8474), and ORP (*r* = 0.8244). Meanwhile, Tur was correlated with RDA2 (*r* = 0.9951), followed by TDS (*r* = 0.8786), Ba (*r* = 0.8636), and EC (*r* = 0.8377, Supplementary Table S2).

The RDA results showed the environmental factors correlated with the seven dominants microbial at the phylum level including TP, Se, Tem, etc (Figure 9). Specifically, Actinobacteria was positively correlated with TN, TP, and Mn. Proteobacteria was negatively correlated with most environmental factors except DO and Cd. In addition, there was a positively correlated between Cyanobacteria, Bacteroidetes, and Verrucomicrobia with EC, TDS, Tur, and Ba (Figure 9). The mantel's test result (Figure 8) showed that the environmental factors include Tem, ORP, TP, and Cd were significantly correlated with the relative abundance of archaea and bacteria community (*P* < 0.05). And, except for the Ba, all environmental factors were highly significantly correlated with the bacteria (*P* < 0.01).

**Figure 10:**
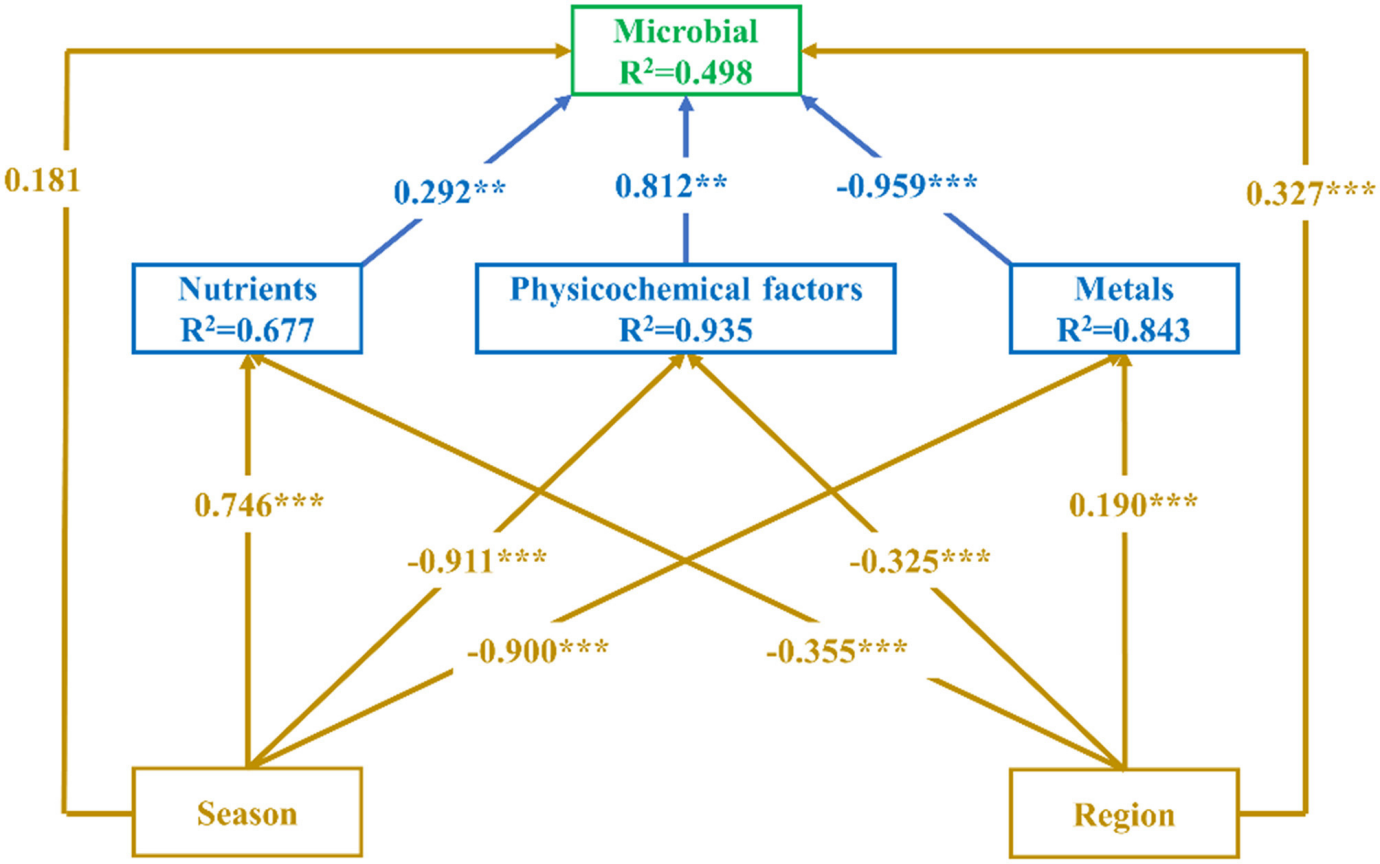
Path diagram for present partial least squares structural equation modeling showing direct and indirect effects of Microbial, Nutrients, Physicochemical factors, metals, Season, and Region. Numbers on the arrows are standard path coefficients that reveal the effect size of the relationship (positive numbers represent positive correlation, negative numbers represent negative correlation). The arrows indicate a one-way directed relationship. Blue arrows indicate relationships between endogenous latent variables, with brown arrows representing the relationship between exogenous and endogenous latent variables. There was variance explained (conditional R^2^) below each latent variable in the model. Significance levels are as follows: **P < 0.01, and ***P < 0.001.

The results of the structural model (Figure 10) revealed that the factor with the highest influence on the OTUs was the metals (path coefficient = −0.959, R^2^ = 0.843, *P* < 0.001), followed by the physicochemical factors (path coefficient = 0.812, R^2^ = 0.935, *P* < 0.01), and the lowest was nutrients (path coefficient = 0.292, R^2^ = 0.621, *P* < 0.01). In addition, there were significant (*P* < 0.001) path coefficients for spatial and physicochemical factors, nutrients, and metals. Although the direct effect of season on the OTUs was not significant, we found that season changed the OTUs by affecting physicochemical factors, metals, and nutrients. Spatial variation had the same significant indirect effects on the OTUs (Supplementary Table S4).

## 4 Discussion

### 4.1 The dominant microbial community in tropical Songtao Reservoir

Our results indicated that Proteobacteria, Actinobacteria, Cyanobacteria, and Bacteroidets accounted for 90% of the total microbial community at the phylum level (Figure 5). This may be related to Proteobacteria, Actinobacteria and Bacteroidetes are all believed to degrade complex organic macromolecules (González et al., 2000; Kirchman, 2002; Zaitlin and Watson, 2006). Previous study showed that Proteobacteria and Actinobacteria were the dominant microbial community in surface water of reservoir which account for more than 50% of the total bacteria (Yu et al., 2014). Earlier research also found that Proteobacteria was a general component in freshwater microbial community, accounting for 60%−70% of all microbial community in rivers, lakes, and reservoir ecosystems (Newton et al., 2011; Parfenova et al., 2013). Similarly, Actinobacteria is also a relatively large group in freshwater (Newton et al., 2011; Ji et al., 2019; Niu et al., 2019). Some studies, for instance, found that Actinobacteria existed in almost all lakes, reservoirs, and rivers, which is related to its involvement in the decomposition of organic matter (Parfenova et al., 2013; Ghai et al., 2014). Bacteroidetes were the most diverse group of bacteria living in mesotrophic and eutrophic water environment (Ligi et al., 2014). Therefore, it is essential for Actinobacteria, Proteobacteria, and Bacteroidetes in determining the microbial composition of water bodies, although the main microbial community differs in freshwater (Jiang et al., 2021). Our results showed that the dominant populations of all samples were similar to other rivers, lakes, and reservoirs in China (Yu et al., 2014; Jiang et al., 2021). However, the composition of microbial communities in this study differed from that of eutrophic rivers and reservoirs, which the dominant microbial were Actinobacteria, Proteobacteria and Bacteroidets (Debroas et al., 2009).

In this study, the relative abundance of Cyanobacteria was high in Songtao Reservoir (Figure 5) than in the subtropical region (Guo et al., 2021; Zhu and An, 2021). Previous studies showed that Cyanobacteria are the simplest and most primitive green autotrophic bacteria, widely distributed in freshwater. Under the influence of global climate change, the abundance of Cyanobacteria in lakes is gradually expanding (Hou et al., 2022). Lürling et al. (2018) found that the Cyanobacteria had a competitive advantage at elevated temperatures. This study was sampled in a tropical region, which may explain the relatively high abundance of cyanobacteria. And, excessive Cyanobacteria are the main feature of blooms (Brooks et al., 2016). Therefore, more attention should be paid to monitoring and controlling Cyanobacteria in the water environment in summer in Songtao Reservoir.

### 4.2 The dynamic distribution characteristics of microbial community and their environmental drivers in Songtao Reservoir

The results indicated that there were significant temporal and spatial differences in microbial community composition across Songtao Reservoir (Figure 6). Specifically, both diversity (Figures 3, 4) and OTUs number (Figure 2) of microbial community showed significant differences among the two seasons and three regions, the PLS-SEM result also indicated that season and region have significant indirect and direct effects on microbial community composition (Figure 10).

#### 4.2.1 Spatial variation in microbial community and the driving factors

We found that there were more species of microbial community in the BS (upstream) of the reservoir than in the NF (midstream) and FJ (downstream), which was the same as the results of Wang et al. (2021). They reported that there was an increase in microbial community diversity upstream of Three Gorges Dam which may be due to microbial community could be dispersed passively via the flow of water along the transfer of material. Liu et al. (2018) found that there was a sudden reduction in microbial diversity at the downstream sites of the dam compared to the upstream sites.

Previous studies indicated that TN and TP played an important role in the growth and reproduction of microbial community (Read et al., 2015; Javed et al., 2021). This may also contribute to the higher microbial species in the BS than in other regions. In this study, the results of environmental factors showed that there were significantly higher concentrations of TN, TP and COD in BS than other two regions (Supplementary Table S1). The RDA result (Figure 9) also showed that TP was the main environmental factor for microbial community composition. Artigas et al. (2012) found that P, an essential nutrient for microbial growth, could be assimilated by the cell membrane. The study of Niu et al. (2019) showed that ${\text{PO}}_{4}^{3-}$ was the main environmental factor affecting bacterial composition. The findings of the mantel's test (Figure 8) also indicated a significant correlation between TP and the community composition of archaea and bacteria.

Moreover, we found that the abundance of Bacteroidetes was significantly higher in the BS than in other regions (Figure 5). This may be related to there were highest concentrations of TDS, Tur, TP, TN, and COD in BS (Supplementary Table S1). Some studies showed that Bacteroidetes is a relatively abundant bacterial community in eutrophic freshwater ecosystems (Trusova and Gladyshev, 2002). Previous study showed that Bacteroidetes reflected the degree of corruption and existed in eutrophic lake (Tang et al., 2009; Ibekwe et al., 2016). This phenomenon further indicated that the water quality in the BS of Songtao Reservoir was poorer than that of the other regions.

#### 4.2.2 Temporal variation in microbial community and the driving factors

Our results also indicated there was a significant temporal difference in microbial community composition (Figure 4). There was a similar finding in other studies (e.g., Liu et al., 2013; El Najjar et al., 2020; Jiang et al., 2021). This may be due to the large difference in water temperature between seasons (Yang et al., 2022). In this study, the RDA result indicated that the temperature was the main driving factor for microbial community composition (Supplementary Table S2), and the mantel's test also proved that the temperature was significantly correlated with bacteria and archaea (Figure 8). The study of Ruhl et al. (2022) also indicated that temperature is a major factor limiting the taxonomic and functional diversity of microbial communities. We found that there were higher microbial community OTU number and diversity significantly higher in winter than in summer (Figure 3). This may be related to the water temperature in the reservoir in tropical areas, e.g., the water temperature in winter can also reach 22°C. The similar results had been found by El Najjar et al. (2020). They found that there were significantly higher total germs of microbial in November to March than from May to August. The study of Sharp et al. (2014) also indicated that a Gaussian relationship was observed for microbial community diversity in the temperature range above 90°C, with diversity peaking at 24°C. Moreover, we found that the levels of TN and TP in the water were higher in winter than in summer in the same region (Supplementary Table S1), which also resulted in a higher number and diversity of microbial community in winter compared to summer.

The seasonal dynamics of the concentrations of elements seemed similar to the changes in microbial diversity and OTUs in winter compared to summer. It has been shown that the appropriate concentrations of essential elements have a growth-promoting effect on microbial community, while the excessive concentrations inhibit their growth and metabolism, namely a phenomenon known as the Homeostasis effect (Calabrese, 1999). We found that the metal concentrations of Songtao Reservoir water body were overall low, which explains the synchrony of the element concentrations with the changes in microbial diversity. In addition, the results of RDA showed that the metal element Se had a greater effect on the composition of microbial communities, which may be due to the fact that selenium could be assimilated and transformed by microbial community to synthesize specific proteins and polysaccharides (Wang et al., 2022). Moreover, the result of mantel's test (Figure 8) and PLS-SEM (Figure 10) also proved the metals were significantly correlated with microbial community composition.

However, in this study, temporal changes in microbial abundance and diversity differed from those in the subtropical region. Jiang et al. (2021) found that the Reads, Shannon, and Chao of bacteria in winter were lower than that in summer in Shanghai, China. And Liu et al. (2013) also found that the number of bacterial bands in the wet season (April to September) was higher than that in the dry season (October to March) in a subtropical river. In addition, our findings of the temporal changes in the relative abundance of the microbial phyla also differed with Jiang et al. (2021). They found a decreasing trend in the relative abundance of Actinobacteria from summer to winter, which was the opposite of the results of the present study.

## 5 Conclusion

In this study, the microbial community abundance in two seasons was quantified by 16S rRNA in Songtao Reservoir, a tropical drinking water reservoir located in the southernmost of China. We validated our hypothesis that the variations of water quality across time and space significantly correlated with the differences in microbial community composition. Briefly, the main species at the phylum level were Actinobacteria, Proteobacteria, Bacteroidetes, and Cyanobacteria in Songtao Reservoir. According to the diversity analysis and cluster analysis, the diversity and number of microbial were significantly higher in winter than in summer, and upstream than in midstream and downstream. The results of microbial community distribution indicated that there was more possibility of the existence of cyanobacterial blooms in summer at Songtao Reservoir. In addition, there was a highly significant correlation between microbial community composition and environmental factors such as temperature, total phosphorus, Se, and Ni. Moreover, the mantel's test showed that the temperature and total phosphorus both affect the community composition in archaea and bacteria. These findings are essential for understanding the distribution characteristics of microbial in drinking water reservoirs in tropical areas and also can provide a reference for the management of reservoir operations to ensure drinking water is safe.

## Data availability statement

The original contributions presented in the study are included in the article/Supplementary material, further inquiries can be directed to the corresponding authors.

## Author contributions

DW: Writing—review & editing, Writing—original draft, Software, Methodology, Investigation, Formal analysis, Data curation. YZ: Writing—original draft, Validation, Software, Methodology. JX: Writing—review & editing, Formal analysis. LM: Writing—review & editing, Software, Methodology. SL: Writing—original draft, Validation, Software, Methodology. BC: Writing—original draft, Data curation, Methodology, Software. QF: Writing—review & editing, Validation, Supervision. ZG: Writing—review & editing, Resources, Funding acquisition, Conceptualization.
