# Supplementary material for: The spatiotemporal variations of microbial community in relation to water quality in a tropical drinking water reservoir, Southmost China
Source: Front Microbiol. 2024 May 6;15:1354784. doi: 10.3389/fmicb.2024.1354784 (PMC11102952; doi:10.3389/fmicb.2024.1354784)
Supplement: Supplementary file 1 [file Table_1.docx]

**Supplementary Materials**

**Supporting Information**

**The spatiotemporal variations of microbial community in relation to water quality in a tropical drinking water reservoir, southmost China**

**Di Wu^b^, Ying Zou^b^, Juan Xiao^b^, Ling Mo^c^, Sovan Lek ^b,d^, Bo Chen^a^, Qiongyao Fu^a^*, Zhiqiang Guo^b^***

^a^ National Health Commission of the People's Republic of China Key Laboratory of Control of Tropical Diseases Control, School of Tropical Medicine, Hainan Medical University, Haikou, Hainan, 571199, China.

^b^ School of Life and Health Sciences, Key Laboratory of Tropical Biological Resources of Ministry of Education, State Key Laboratory of Marine Resource Utilization in South China Sea, Hainan University, Haikou, 570228, China.

^c^ Hainan Research Academy of Environmental Sciences, Haikou 571126, China.

^d^ Laboratoire Evolution & Diversit´e Biologique, Universit´e Paul Sabatier, 118 route de Narbonne, Toulouse c´edex 4 31062, France.

*Corresponding author: Qiongyao Fu (e-mail: [fuqy2020@163.com](mailto:fuqy2020@163.com)); Zhiqiang Guo (e-mail: [guozq@hainanu.edu.cn](mailto:guozq@hainanu.edu.cn)).

**Contents of Supporting Information**

Figure for ANOSIM of microbial abundances.............................................................Fig. S1

Figure for Lefse of microbial abundances at phylum level…....................................Fig. S2

Figure for Lefse of microbial abundances at genus level...........................................Fig. S3

Table for environmental factors..............................................................................Table S1

Table for correlation coefficient of RDA................................................................Table S2

Table for reliability and validity of PLS-SEM........................................................Table S3

Table for specific indirect effects of PLS-SEM......................................................Table S4


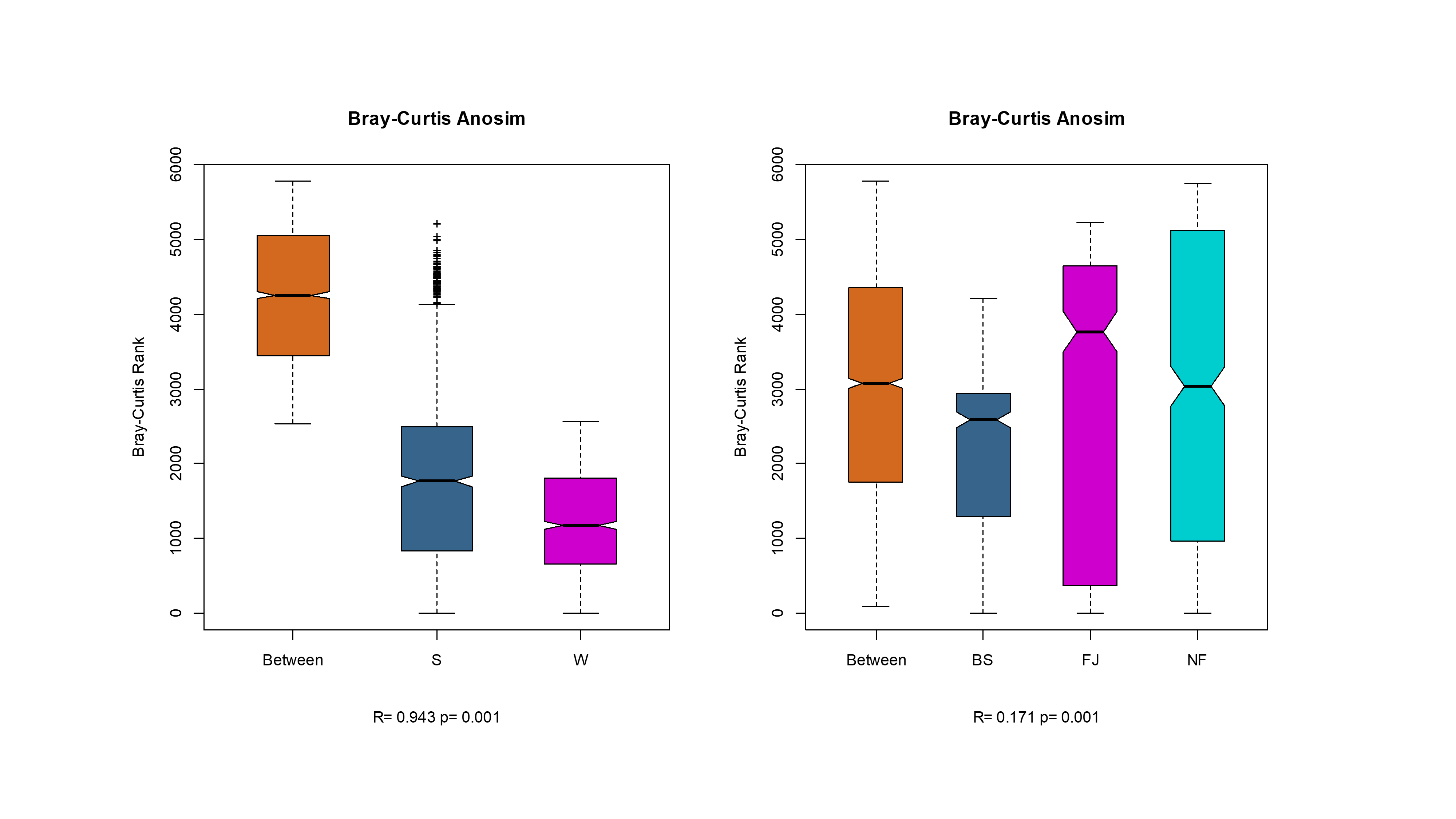


**Fig. S1** The ANOSIM of spatiotemporal microbial abundances in Songtao Reservoir


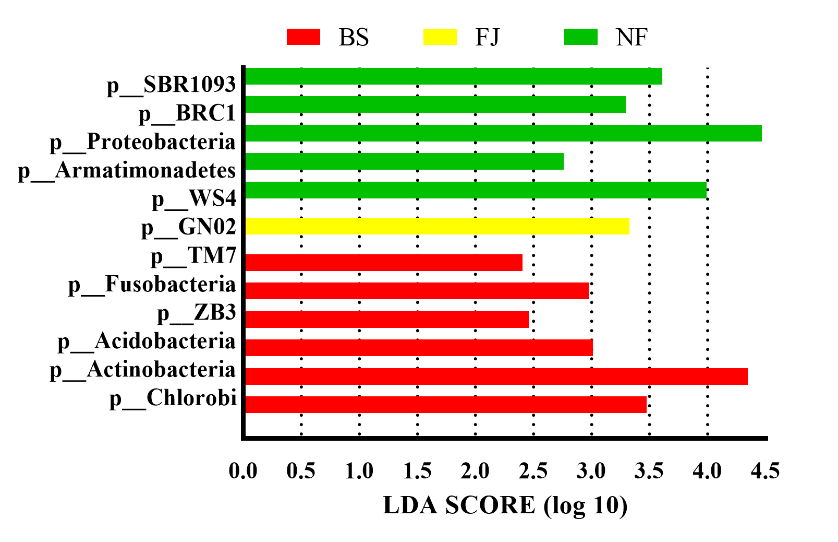

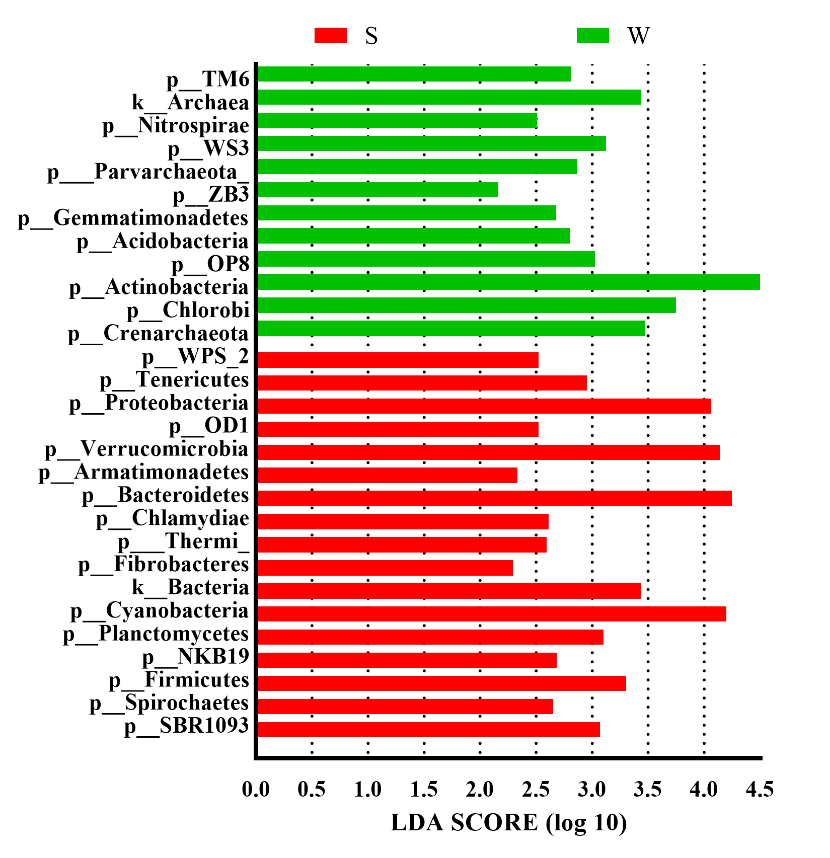


**Fig. S2** Comparison of different spatiotemporal microbial abundances in Songtao Reservoir at phylum level (LDA Score > 2.0).


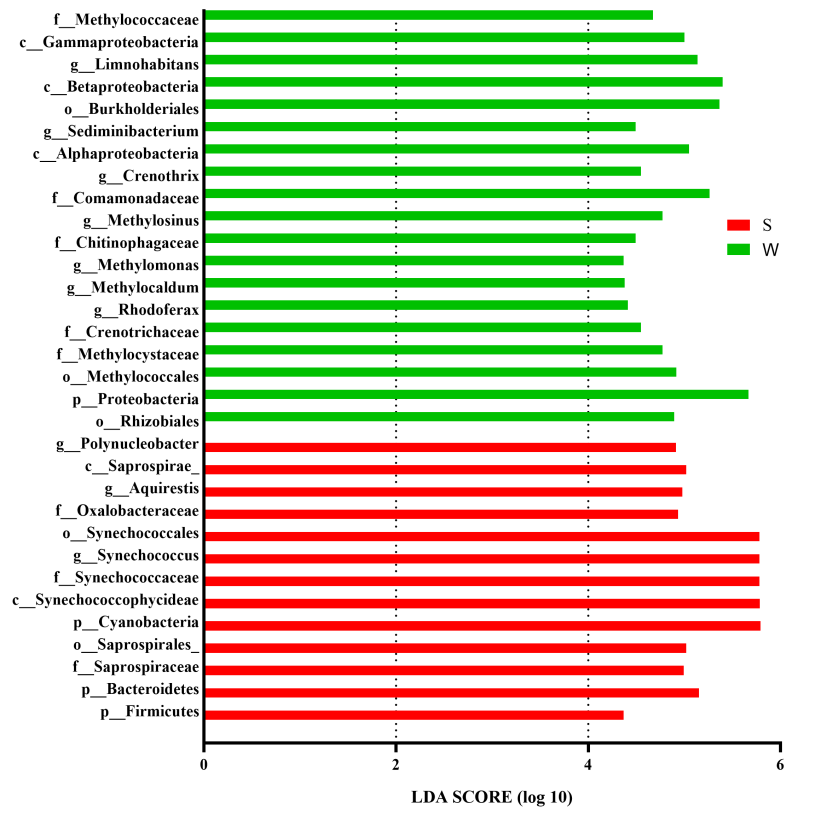


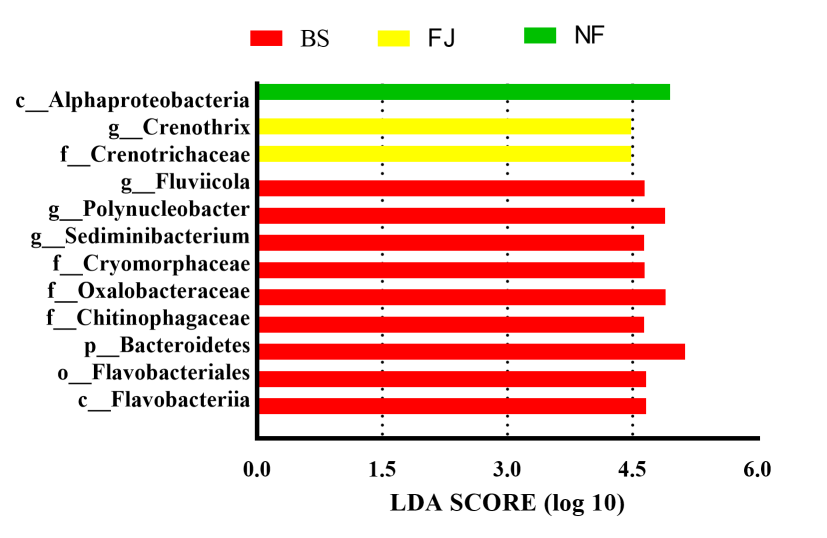


**Fig. S3** Comparison of different spatiotemporal microbial abundances in Songtao Reservoir at genus level (LDA Score > 4.0).

**Table S1**

The results of environmental factors.

|  | NF | | FJ | | BS | |
| --- | --- | --- | --- | --- | --- | --- |
|  | Summer | Winter | Summer | Winter | Summer | Winter |
| pH | 8.16-8.45 | 7.09-7.20 | 8.07-8.39 | 6.92-7.20 | 8.32-8.49 | 7.26-7.81 |
| EC  (mS cm^-1^) | 82.4-99.6 | 72.0-74.8 | 86.7-89.0 | 66.7-70.8 | 113.1-151.6 | 77.6-81.8 |
| DO  (mg L^-1^) | 7.05-8.07 | 5.17-8.79 | 7.05-8.07 | 7.30-7.75 | 8.08-9.17 | 4.72-6.46 |
| T (℃） | 31.4-32.3 | 22.1-22.6 | 32.1-32.7 | 21.5-22.9 | 32.1-33.1 | 22.2-23.5 |
| TDS  (g L^-1^） | 0.053-0.065 | 0.049-0.051 | 0.054-0.057 | 0.047-0.048 | 0.074-0.096 | 0.053-0.055 |
| ORP (mV) | 81.0-133.8 | 178.9-225.4 | 85.5-189.5 | 170.0-234.1 | 120.1-140.0 | 112.0-146.6 |
| Tur (NTU) | 2.54-2.80 | 2.02-2.86 | 2.04-2.78 | 1.69-2.81 | 3.29-8.37 | 2.34-3.52 |
| TN  (mg L^-1^) | 0.09-0.41 | 0.17-0.34 | 0.10-0.11 | 0.17-0.28 | 0.13-0.23 | 0.28-1.28 |
| TP  (mg L^-1^) | 0.006-0.015 | 0.023-0.036 | 0.006-0.011 | 0.035-0.041 | 0.013-0.032 | 0.041-0.045 |
| COD  (mg L^-1^) | 1.46-1.70 | 1.01-1.17 | 1.30-1.83 | 1.63-1.97 | 1.88-2.61 | 1.81-3.20 |
| Cr  (μg L^-1^) | 0.289-0.560 | 0.005-0.106 | 1.105-1.683 | 0.019-0.059 | 0.437-0.815 | 0.000-0.072 |
| Mn  (μg L^1^) | 6.558-6.666 | 56.402-119.208 | 9.055-11.328 | 31.940-197.475 | 5.080-21.280 | 5.203-126.595 |
| Ni  (μg L^-1^) | 1.307-1.504 | 0.306-0.367 | 1.441-2.229 | 0.211-0.260 | 1.712-1.869 | 0.226-0.478 |
| Cu  (μg L^1^) | 0.472-0.589 | - | 0.709-2.424 | - | 0.851-1.339 | - |
| Se  (μg L^-1^) | 4.921-5.46 | 1.679-2.051 | 5.302-5.442 | 2.212-2.738 | 5.054-5.661 | 1.898-1.978 |
| Cd  (μg L^1^) | 0.005-0.023 | 0.001-0.018 | 0.010-0.016 | 0.001-0.002 | 0.008-0.010 | 0.001-0.004 |
| Ba  (μg L^-1^) | 11.751-56.560 | 34.473-60.160 | 55.153-63.570 | 60.545-71.664 | 13.892-58.373 | 43.175-54.821 |

Note: - denotes the values were under detection limiting.

**Table S2**

Correlation coefficient between environmental factors and microbial community.

|  | RDA1 | RDA2 | r^2^ | *P* values |
| --- | --- | --- | --- | --- |
| Tem | -0.9135 | 0.4067 | 0.8107 | 0.001 |
| DO | -0.9933 | -0.1160 | 0.3678 | 0.001 |
| C | -0.5462 | 0.8377 | 0.3428 | 0.001 |
| TDS | -0.4776 | 0.8786 | 0.3063 | 0.001 |
| pH | -0.8567 | 0.5158 | 0.6723 | 0.001 |
| ORP | 0.8244 | -0.5660 | 0.1413 | 0.002 |
| Tur | -0.0987 | 0.9951 | 0.2583 | 0.001 |
| TP | 1.0000 | -0.0101 | 0.7687 | 0.001 |
| TN | 0.9949 | 0.1011 | 0.4070 | 0.001 |
| Cr | -0.8669 | 0.4985 | 0.6084 | 0.001 |
| Mn | 0.8496 | -0.5274 | 0.3502 | 0.001 |
| Ni | -0.8931 | 0.4498 | 0.7267 | 0.001 |
| Cu | -0.8474 | 0.5310 | 0.5388 | 0.001 |
| Se | -0.9206 | 0.3905 | 0.8370 | 0.001 |
| Cd | -0.9845 | -0.1755 | 0.3388 | 0.001 |
| Ba | -0.5042 | 0.8636 | 0.1066 | 0.004 |

**Table S3**

The construct reliability and validity of PLS-SEM.

|  | Cronbach's Alpha | rho_A | CR | AVE |
| --- | --- | --- | --- | --- |
| Metals | 0.959 | 0.961 | 0.968 | 0.860 |
| Nutrients | 0.550 | 0.958 | 0.758 | 0.655 |
| Physicochemical factors | 0.928 | 0.956 | 0.947 | 0.818 |

**Table S4**

The specific indirect effects of PLS-SEM

|  | path coefficients | *P* values |
| --- | --- | --- |
| season - heavy metals - microbial | 0.863 | 0.000 |
| season - physicochemical factors - microbial | -0.740 | 0.012 |
| region - heavy metals - microbial | -0.182 | 0.000 |
| region - nutrients - microbial | -0.104 | 0.030 |
| season - nutrients - microbial | 0.218 | 0.018 |
| region - physicochemical factors - microbial | -0.264 | 0.013 |
